# Evaluation of seven tumour markers in pleural fluid for the diagnosis of malignant effusions

**DOI:** 10.1038/sj.bjc.6690807

**Published:** 1999-11

**Authors:** M Miédougé, P Rouzaud, G Salama, M-C Pujazon, C Vincent, M-A Mauduyt, J Reyre, P Carles, G Serre

**Affiliations:** Department of Biology and Pathology of the Cell, INSERM CJF 96-02/IFR30, Toulouse Purpan School of Medicine, University of Toulouse III, Toulouse Cedex 31059, France; Department of Internal Medicine, Purpan Hospital, Toulouse Cedex 31059, France

**Keywords:** tumour markers, pleural effusions, carcinomas

## Abstract

Carcinoembryonic antigen (CEA), carbohydrate antigens 15–3, 19–9 and 72–4 (CA 15–3, CA 19–9 and CA 72–4), cytokeratin 19 fragments (CYFRA 21–1), neuron-specific enolase (NSE) and squamous cell carcinoma antigen (SCC) were evaluated in pleural fluid for the diagnosis of malignant effusions. With a specificity of 99%, determined in a series of 121 benign effusions, the best individual diagnostic sensitivities in the whole series of 215 malignant effusions or in the subgroup of adenocarcinomas were observed with CEA, CA 15–3 and CA 72–4. As expected, a high sensitivity was obtained with SCC in squamous cell carcinomas and with NSE in small-cell lung carcinomas. CYFRA and/or CA 15–3 were frequently increased in mesotheliomas. Discriminant analysis showed that the optimal combination for diagnosis of non-lymphomatous malignant effusions was CEA + CA 15–3 + CYFRA + NSE: sensitivity of 94.4% with an overall specificity of 95%. In malignant effusions with a negative cytology, 83.9% were diagnosed using this association. The association CYFRA + NSE + SCC was able to discriminate adenocarcinomas from small-cell lung cancers. Regarding their sensitivity and their complementarity, CEA, CA 15–3, CYFRA 21–1, NSE and SCC appear to be very useful to improve the diagnosis of malignant pleural effusions. © 1999 Cancer Research Campaign


*British Journal of Cancer* (1999) **81**(5), 1059–1065
© 1999 Cancer Research Campaign
Article no. bjoc.1999.0807

### Evaluation of seven tumour markers in pleural fluid for the diagnosis of malignant effusions

**M Miédougé[1], P Rouzaud[2], G Salama[1], M-C Pujazon[2], C Vincent[1], M-A Mauduyt[1], J Reyre[1], P Carles[2] and G Serre[1]**

[1]Department of Biology and Pathology of the Cell, INSERM CJF 96-02/IFR30, Toulouse Purpan School of Medicine, University of Toulouse III, 31059 Toulouse Cedex, France; [2]Department of Internal Medicine, Purpan Hospital, 31059 Toulouse Cedex, France

**Summary** Carcinoembryonic antigen (CEA), carbohydrate antigens 15–3, 19–9 and 72–4 (CA 15–3, CA 19–9 and CA 72–4), cytokeratin 19 fragments (CYFRA 21–1), neuron-specific enolase (NSE) and squamous cell carcinoma antigen (SCC) were evaluated in pleural fluid for the diagnosis of malignant effusions. With a specificity of 99%, determined in a series of 121 benign effusions, the best individual diagnostic sensitivities in the whole series of 215 malignant effusions or in the subgroup of adenocarcinomas were observed with CEA, CA 15–3 and CA 72–4. As expected, a high sensitivity was obtained with SCC in squamous cell carcinomas and with NSE in small-cell lung carcinomas. CYFRA and/or CA 15–3 were frequently increased in mesotheliomas. Discriminant analysis showed that the optimal combination for diagnosis of non-lymphomatous malignant effusions was CEA + CA 15–3 + CYFRA + NSE: sensitivity of 94.4% with an overall specificity of 95%. In malignant effusions with a negative cytology, 83.9% were diagnosed using this association. The association CYFRA + NSE + SCC was able to discriminate adenocarcinomas from small-cell lung cancers. Regarding their sensitivity and their complementarity, CEA, CA 15–3, CYFRA 21–1, NSE and SCC appear to be very useful to improve the diagnosis of malignant pleural effusions. © 1999 Cancer Research Campaign

**Keywords:** tumour markers; pleural effusions; carcinomas

Malignancies represent one of the main aetiologies of pleurisies. Carcinoma of any organ can metastasize to the pleura but the cancers most concerned are lung and breast carcinomas, lymphomas and, less frequently, digestive and ovary carcinomas (Serre et al, 1990; Fenton and Richardson, 1995; Sahn, 1988). Pleural mesothelioma also occurs rarely but its frequency is increasing (Peto et al, 1995).

Since in about 40% of malignant effusions cytological examination of pleural fluid does not allow the detection of tumour cells (Loddenkemper and Boutin, 1993; Fenton and Richardson, 1995; Harris et al, 1995; Sahn, 1988), several authors investigated the assay of tumour markers in pleural fluid in order to improve the diagnostic performances. Carcinoembryonic antigen (CEA) has been studied the most and has shown a diagnostic sensitivity of about 50–60% (Rapellino et al, 1990). Nevertheless, for a particular carcinoma the use of a single marker appeared insufficient because it is not always expressed and, in addition, no tumour marker has a spectrum wide enough to detect all types of malignancies. Thus, many studies emphasized the association of CEA with various tumour markers such as carbohydrate antigens 15–3, 19–9 and 72–4 (CA 15–3, CA 19–9 and CA 72–4) (Ferroni et al, 1990; Villena et al, 1996). The best results were logically obtained in adenocarcinomas since serum CA 15–3 is proposed for the management of breast adenocarcinomas (Soletormos et al, 1996), CA 72–4 for gastric and ovarian adenocarcinomas (Guadagni et al, 1995) and CA 19–9 for digestive adenocarcinomas (Grem, 1997). CYFRA 21–1, a serum assay for soluble fragments of cytokeratin 19, was recently proposed for the diagnosis and the follow-up of non-small-cell lung carcinomas (Pujol et al, 1993; Plebani et al, 1995) but also for squamous cell carcinomas of the head and neck (Doweck et al, 1995) and uterine cervix (Callet et al, 1998) and lastly in bladder cancer (Morita et al, 1997). Moreover, we recently described high values of this marker in the pleural fluid of patients with mesothelioma (Salama et al, 1998).

Within these numerous studies of tumour markers in malignant effusions, the use of various non-equivalent assays and the heterogeneity or the small size of the samples of patients make comparison of the results difficult and generalization impossible. Furthermore, depending on the studies, statistical analyses and pleural threshold determinations varied greatly (Rapellino et al, 1990); the worst solution, i.e. the use of serum thresholds, being chosen in numerous cases. Indeed, at the same level of specificity, pleural and serum thresholds are different, as shown for CEA (Rittgers et al, 1978, Romero et al, 1996) and also for CYFRA (Toumbis et al, 1996). In patients with malignant effusions, pleural values of markers are higher than serum values and the greater sensitivity of the pleural assay for the diagnosis of malignancy was demonstrated for several markers such as CEA (Rittgers et al, 1978; Klockars et al, 1980; Asseo and Tracopoulos, 1982; Rapellino et al, 1990; Romero et al, 1996), CA 15–3 (Rapellino et al, 1990) or neuron-specific enolase (NSE) (Menard et al, 1993).

For all these reasons, in the present study, we evaluated in pleural fluid a large panel of tumor markers including CEA, CA 15–3, CA 72–4 and CA 19–9, regarding their interest in adenocarcinomas, and CYFRA, for its interest in squamous cell carcinomas and mesotheliomas. We also analysed squamous cell carcinoma antigen (SCC) and NSE, serum markers of squamous cell carcinomas (Niklinski and Furman, 1995; Callet et al, 1998) and small-cell lung carcinomas (Niklinski and Furman, 1995; Plebani et al, 1995) respectively. Our objective was to determine the optimal panel able to improve the diagnosis of malignant effusions, particularly in cytologically negative effusions. This study is, to our knowledge, the first where all these markers were evaluated together and in such a large series of patients.

*Received 6 January 1999*
*Revised 12 April 1999*
*Accepted 22 April 1999*

*Correspondence to:* G Serre, Laboratoire de Biologie Cellulaire et Cytologie, CHU Purpan, Place du Dr Baylac, 31059 Toulouse Cedex, France. E-mail: serre.g@chu-toulouse.fr

### MATERIALS AND METHODS

### Patients

We retrospectively studied 336 pleural effusions collected from patients of the Department of Internal Medicine of Purpan Hospital in Toulouse, France (Table 1). Part of this series was constituted of 156 available effusions (out of 199) used in a previous report to evaluate pleural CYFRA in the diagnosis of malignant effusions (Salama et al, 1998). This series was completed by 180 pleural effusions with perfectly defined aetiology, obtained from July 1996 to December 1997.

In a control group of 121 patients (62.2% men, aged from 10 to 95 years, median 71 years), a benign disease was diagnosed and confirmed by follow-up and/or efficiency of a specific treatment. Malignant pleural involvement was ascertained in 215 patients (52.1% men, aged from 19 to 89 years, median 65 years), by the presence of malignant cells in pleural fluid and/or in pleural biopsy (blind needle biopsy or biopsy under thoracoscopy). These two groups were representative of the main causes of pleurisies (Serre et al, 1990; Fenton and Richardson, 1995; Villena et al, 1996; Sahn, 1988). Breast and lung cancer were the most frequent causes of malignant pleural involvement. Malignant effusions of unknown primary site were frequent in our recruitment (46 out of 215, i.e. 21%) in agreement with the prospective study of Villena et al (1996) (17 out of 65, i.e 26%).

**Table 1** Sample of patients

| **Diagnosis** | ***n*** |
|---|---|
| Carcinomas | **183** |
| Adenocarcinomas | 154 |
| Breast | 50 |
| Lung | 38 |
| Digestive | 11 |
| Ovary | 6 |
| Other organs[a] | 3 |
| Unknown primary site | 46 |
| Small-cell lung carcinomas | 13 |
| Squamous cell carcinomas | 11 |
| Lung | 4 |
| Digestive | 3 |
| Other sites[b] | 4 |
| Other carcinomas[c] | 5 |
| Mesotheliomas | **11** |
| Non-epithelial malignancies | **21** |
| Lymphomas/leukaemias | 18 |
| Sarcomas | 3 |
| Benign | **121** |
| Cardiac failure | 39 |
| Parapneumonic | 26 |
| Tuberculosis | 21 |
| Systemic diseases[d] | 9 |
| Other diseases[e] | 26 |

[a]Including uterus (2) and kidney (1) adenocarcinoma. [b]Including skin (1), uterus (2) and head and neck (1) squamous cell carcinoma. [c]Including bladder carcinoma (3), and digestive and lung carcinoid tumour (2). [d]Including systemic lupus erythematosus (3), rheumatoid arthritis (2), Gougerot-Sjögren's syndrome (1), scleroderma (1), Sharp's syndrome (1) and Still's disease (1). [e]Including post-traumatic (9), pulmonary embolism (5), benign asbestosis (3), chylothorax (1), lung fibrosis (1), cirrhosis (5), nephrotic syndrome (1) and sarcoidosis (1).

### Cytological analysis and tumour marker assay

Pleural fluid was obtained by thoracocentesis, collected in sterile tubes without anticoagulant and rapidly brought to the laboratory. After performing a cell count of the sample of pleural fluid with a haemocytometer, optimal dilution was carried out in order to obtain 300 nucleated cells $\mu l^{-1}$ and several samples of 0.7 ml were cytocentrifuged at 700 rpm for 17 min in a Cytospin 2™ (Shandon Ltd, Cheshire, UK). Air-dried slides were stained with the May–Grünwald–Giemsa method, and absolute ethanol-fixed slides were stained with the Papanicolaou method, for morphological examination. Periodic acid-Schiff and Alcian blue (pH 2.5) cytochemical reactions and immunocytochemical analysis, using a panel of monoclonal antibodies to CEA, cytokeratins, vimentin, epithelial membrane antigen, desmin, B72–3 and Ber EP4, were also performed on the slides to aid the characterization of suspect and malignant cells (Serre et al, 1990; Daste et al, 1991). Part of the sample was centrifuged and the supernatant aliquoted and stored at –80°C until tumour markers were assayed with commercial enzyme immunoassays: CEA, CA 19–9 (Axsym System®, Abbott Diagnostic, France), SCC (IMX System®, Abbott Diagnostic, France), CYFRA and CA 72–4 (Enzymun-Test®, Boehringer Mannheim, France), CA 15–3 and NSE (CPE-CA 15–3® and CPE-NSE®, Cis Bio International, France). The tumour markers were assayed in duplicate and dilutions were carried out, if necessary, with the appropriate diluent as recommended by the manufacturer.

**Table 2** Median and range of tumour markers in benign and malignant effusions

| **Tumour markers** | **Benign effusions *n*=121** | **Malignant effusions *n*=215** | ***P*** |
|---|---|---|---|
| CEA (ng ml$^{-1}$) | 1 (0–6.2) | 17.2 (0.2–12785) | < 0.00001 |
| CA 72-4 (U ml$^{-1}$) | 2 (1.6–4.5) | 21.8 (1.6–20000) | < 0.00001 |
| CA 15-3 (U ml$^{-1}$) | 13.5 (2–50.8) | 68.9 (0.5–3581) | < 0.00001 |
| CA 19-9 (U ml$^{-1}$) | 1.2 (0–550.3) | 5.9 (0–480000) | < 0.00001 |
| CYFRA (ng ml$^{-1}$) | 16.3 (0–188.4) | 107.4 (1.8–12796.5) | < 0.00001 |
| NSE (ng ml$^{-1}$) | 3.3 (0.1–375) | 7.3 (0–408) | < 0.00001 |
| SCC (ng ml$^{-1}$) | 1.6 (0–38.4) | 2.2 (0–2420) | 0.22 |

### Data analysis

Descriptive statistics and discriminant analysis were performed using STATISTICA 5.1 (StatSoft, Inc, Tulsa, OK, USA). The median and range of each marker were determined in the groups of benign and malignant effusions. Differences between groups were tested with Mann–Whitney's *U*-test. Pleural thresholds were defined, in the group of benign effusions, at various levels of diagnostic specificities. Corresponding levels of sensitivities were determined in various groups of malignant effusions. Receiver Operating Characteristic curves were also constructed for each marker and the areas under the curves were compared (Hanley and McNeil, 1983). Discriminant analysis constitutes a powerful tool to choose, among many parameters, the optimal combination of two or more of them which is able to separate groups in a population. This approach was previously applied to the evaluation of tumour markers, particularly to distinguish small-cell lung cancer from non-small-cell lung cancer (Paone et al, 1996). Thus, we performed discriminant analysis, after logarithmic transformation of the values, to determine which markers are significantly able to

*© 1999 Cancer Research Campaign*

**Table 3** Thresholds and diagnostic sensitivity (percentage) of the seven tumour markers in the various groups of malignant effusions

| Thresholds[a] | *n* | CEA 6 ng ml$^{-1}$ | CA 15-3 36.2 U ml$^{-1}$ | CYFRA 163 ng ml$^{-1}$ | CA 19-9 219 U ml$^{-1}$ | CA 72-4 3.4 U ml$^{-1}$ | SCC 27 ng ml$^{-1}$ | NSE 18.1 ng ml$^{-1}$ |
|---|---|---|---|---|---|---|---|---|
| All malignant effusions | 215 | 60.0 | 63.7 | 42.8 | 20.9 | 68.4 | 5.6 | 18.1 |
| Non-lymphomatous | 197 | 65.5 | 68.5 | 46.7 | 22.8 | 73.1 | 6.1 | 18.8 |
| Carcinomas | 183 | 70.5 | 71.0 | 47.0 | 24.6 | 78.7 | 6.6 | 18.0 |
| Adenocarcinomas | 154 | 75.3 | 77.9 | 52.6 | 26.6 | 85.1 | 3.2 | 12.3 |
| Squamous cell carcinomas | 11 | 54.5 | 63.6 | 27.3 | 18.2 | 72.7 | 63.6 | 9.1 |
| Small-cell lung carcinomas | 13 | 53.8 | 7.7 | 7.7 | 15.4 | 30.8 | 0.0 | 92.3 |
| Miscellaneous | 5 | 0.0 | 40.0 | 20.0 | 0.0 | 20.0 | 0.0 | 20.0 |
| Mesotheliomas | 11 | 0.0 | 45.5 | 54.5 | 0.0 | 0.0 | 0.0 | 9.1 |
| Sarcomas | 3 | 0.0 | 0.0 | 0.0 | 0.0 | 0.0 | 0.0 | 100.0 |
| Lymphomas | 18 | 0.0 | 11.1 | 0.0 | 0.0 | 16.7 | 0.0 | 11.1 |

[a]Thresholds were chosen to determine a diagnostic specificity of 99% (except for NSE: 97.5%).

**Table 4** Diagnostic sensitivity (percentage) of the association CEA, CA 15-3, CYFRA and NSE in the various groups of malignant effusions considering all the effusions and only the cytologically negative effusions, with an overall diagnostic specificity of 95%

| | All effusions | | Cytologically negative effusions | |
|---|---|---|---|---|
| | *n* | Sensitivity | *n* | Sensitivity |
| All malignancies | 215 | 88.4 | 35 | 74.3 |
| Non-lymphomatous | 197 | 94.4 | 31 | 83.9 |
| Carcinomas | 183 | 95.6 | 23 | 87.0 |
| Adenocarcinomas | 154 | 97.4 | 19 | 94.7 |
| Squamous cell carcinomas | 11 | 81.8 | 2 | 50.0 |
| Small cell lung carcinomas | 13 | 100.0 | 0 | – |
| Miscellaneous | 5 | 60.0 | 2 | 50.0 |
| Mesotheliomas | 11 | 72.7 | 7 | 71.4 |
| Sarcomas | 3 | 100.0 | 1 | 100.0 |
| Lymphomas | 18 | 22.2 | 4 | 0.0 |

separate benign from malignant effusions. Then the diagnostic sensitivity and specificity of their association were evaluated. We also searched for a discriminant combination of markers able to predict the histological type and the primary site of the tumour of origin. Differences were considered significant for $P \leq 0.01$.

### RESULTS

### Tumour markers in benign and malignant effusions

The median and range of each marker in benign and malignant effusions are summarized in Table 2. Except for SCC, the distribution of the values appeared significantly higher in malignant than in benign effusions. Considering the subclasses of benign effusions, we observed a significant but moderate elevation of NSE and CA 15–3 in the group of patients with tuberculosis and very high values for NSE in the two patients with rheumatoid arthritis.

### Diagnostic indexes

The performance of each marker at various levels of specificity was analysed by comparing areas under the receiver operating characteristic curves (Figure 1). The areas of CA 72–4, CEA, CA 15–3 and CYFRA appeared similar and significantly higher than those of CA 19–9 and NSE, themselves greater than the area of

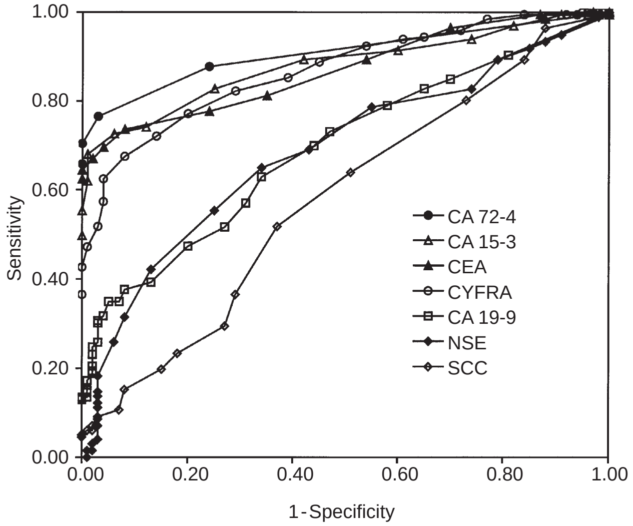

**Figure 1** Receiver operating characteristic curves for each tumour marker considering the group of benign effusions for specificity and the group of non-lymphomatous effusions for sensitivity. The areas under the curves were similar for CA 72-4, CA 15-3, CEA and CYFRA (0.884, 0.880, 0.871 and 0.868 respectively), significantly higher than the areas of CA 19-9 and NSE (0.714 and 0.698) themselves significantly higher than the area of SCC (0.589)

**Table 5** Prediction of the histological type of malignancies using discriminant analysis[a]

| | Predicted origin (number) | | | | |
|---|---|---|---|---|---|
| | Adenocarcinomas | Small-cell lung carcinomas | Squamous cell carcinomas | Mesotheliomas | Correct classification |
| Observed origin (number) | | | | | |
| Adenocarcinomas (154) | 148 | 1 | 4 | 1 | 96.1% |
| Small-cell lung carcinomas (13) | 1 | 12 | 0 | 0 | 92.3% |
| Squamous cell carcinomas (11) | 4 | 0 | 7 | 0 | 63.6% |
| Mesotheliomas (11) | 9 | 0 | 0 | 2 | 18.2% |
| Correct prediction | 91.4% | 92.3% | 63.6% | 66.6% | Total: 89.4% |

[a]CYFRA, SCC and NSE were significantly contributive ($P < 0.00001$) unlike CEA, CA 72-4, CA 15-3 and CA 19-9 ($P > 0.01$).

**Table 6** Prediction of the organ of origin of adenocarcinomas using discriminant analysis[a]

| | Predicted origin (number) | | | | |
|---|---|---|---|---|---|
| | Breast | Lung | Digestive | Ovary | Correct classification |
| Observed origin (number) | | | | | |
| Breast (50) | 40 | 10 | 0 | 0 | 80.0% |
| Lung (38) | 13 | 19 | 4 | 2 | 50.0% |
| Digestive (11) | 1 | 3 | 7 | 0 | 63.6% |
| Ovary (6) | 3 | 1 | 0 | 2 | 33.3% |
| Correct prediction | 70.2% | 57.6% | 63.6% | 50% | Total: 64.8% |

[a]CEA, CA 15-3, CA 19-9 and NSE were significantly contributive ($P < 0.001$) unlike CYFRA, CA 72-4 and SCC ($P > 0.1$).

SCC. Thresholds determined at a diagnostic specificity of 99% (except for NSE for which the 97.5% specificity threshold yielded a clearly higher sensitivity) and the corresponding diagnostic sensitivities in the various histological groups of malignant effusions are listed in Table 3. In carcinomas, the best results were observed for CEA, CA 72–4, CA 15–3 and CYFRA while, in mesotheliomas, only CYFRA and CA 15–3 showed high sensitivities. NSE exhibited a very high sensitivity in small-cell lung carcinomas and in some rare malignancies such as sarcomas, while SCC was only seen to be useful in the diagnosis of squamous cell carcinomas.

### Combinations of markers

Discriminant analysis was used to identify a minimum number of markers able to classify benign versus non-lymphomatous malignant effusions. CEA, CA 15–3, CYFRA and NSE appeared significantly discriminant ($P < 0.002$). By contrast, CA 19–9, SCC but also CA 72–4, despite its high individual sensitivity, did not appear significantly contributive ($P > 0.1$). Table 4 reports the sensitivities of the panel (CEA + CA 15–3 + CYFRA + NSE) in various groups of malignant effusions, with the 99% specificity thresholds for CEA, CA 15–3 and CYFRA and the 97.5% specificity threshold for NSE. A result was considered positive if at least one marker was above its threshold. For the association, the high levels of individual specificity led to an overall specificity remaining high at 95% while we noted a significant improvement in overall sensitivity in non-lymphomatous malignancy at 94.4%.

### Tumour markers in cytologically negative effusions

We applied the same approach to cytologically negative effusions whose neoplastic origin was ascertained by more invasive methods such as blind needle biopsy or biopsy under thoracoscopy. The diagnostic performance of CEA, CA 15–3, CYFRA and NSE remained very convincing in this situation since it allowed the detection of 26 out of 31 (83.9%) cytologically negative non-lymphomatous malignant effusions (Table 4).

### Tumour markers and origin of the primary site

The ability of tumour markers to predict the main histological types or the primary site of the tumour was investigated by discriminant analysis. NSE, SCC and CYFRA appeared to be the most contributive to differentiate adenocarcinomas, small-cell lung carcinomas, squamous cell carcinomas and mesotheliomas. A correct prediction was achieved in 89.4% of the cases, with the best results in adenocarcinomas and small-cell lung carcinomas (Table 5). Mesotheliomas were greatly misclassified since the association of high CYFRA and/or CA 15–3 values to low levels of CEA found in mesotheliomas was also seen in some adenocarcinomas. Concerning the primary site of adenocarcinomas, CEA, CA 15–3, CA 19–9 and NSE were the most significant markers but only 64.8% of adenocarcinomas with a known primary site were correctly classified (Table 6).

### DISCUSSION

Seven tumour markers were evaluated in pleural fluid for the diagnosis of malignant effusions. Despite its utility as a serum marker

 © 1999 Cancer Research Campaign for the management of ovarian cancer, carbohydrate antigen 125 was not included in our study because of its poor diagnostic specificity in pleural effusions (Ferroni et al, 1990; Rapellino et al, 1990; Zeimet et al, 1996). Since a high diagnostic specificity is essential for the clinical use of tumour markers, we first defined highly specific thresholds for each marker. Empyemas were excluded from our series since, in agreement with several reports (Klockars et al, 1980; Garcia-Pachon et al, 1997; Villena et al, 1998) we previously observed high false-positive rates for CEA, but also for CYFRA (Salama et al, 1998) in these fluids. This exclusion is not prejudicial because cytological and bacteriological analyses of pleural fluid easily identify these effusions and overall, malignancy is very rarely associated with this clinical presentation (Sahn, 1988). The very high and isolated levels of NSE found in the two cases of rheumatoid arthritis are in perfect agreement with the observation of Nyberg et al (1996) who described such an increase in 14 out of 17 patients.

In malignant effusions, comparative analysis highlighted the similar diagnostic performances of CEA, CA 72–4, CA 15–3 and CYFRA, clearly superior to those of CA 19–9, NSE and SCC. The diagnostic sensitivity for pleural CEA was concordant with those described in previous studies, confirming its utility in carcinomas (Rapellino et al, 1990) and its non-expression in mesotheliomas (Mezger et al, 1994). Few studies on pleural fluid have integrated CA 72–4 (Ferroni et al, 1990, Villena et al, 1996) and our results confirmed the high sensitivity of CA 72–4 in all types of adenocarcinomas, but we also noted high levels in squamous cell carcinomas. Similarly, as previously described (Ferroni et al, 1990, Romero et al, 1996; Villena et al, 1996), we observed high values of CA 15–3 in adenocarcinomas and not only in those of breast origin. Furthermore, compared to CEA and CA 72–4, CA 15–3 showed an atypical pattern in mesotheliomas, with a sensitivity of 45.5%, in agreement with the results of Villena et al (1996). The data published for pleural CYFRA are more controversial, notably concerning the thresholds and the overall sensitivity which is described as either higher (Satoh et al, 1995) or lower than the sensitivity of CEA (Romero et al, 1996). Confirming our prior study (Salama et al, 1998), we observed similar sensitivities for pleural CEA and pleural CYFRA but above all, a high sensitivity for pleural CYFRA in mesotheliomas.

The most meaningful part of the present study was the evaluation of various combinations of these markers. The optimal panel was CEA + CA 15–3 + CYFRA + NSE. It led to very high diagnostic performance: sensitivity of 88.4% for all the malignant effusions and 94.4% for non-lymphomatous effusions, with a specificity of 95%. Therefore, these four markers showed a very large diagnostic spectrum and only a few types of carcinomas in our series remained undetectable (kidney adenocarcinoma, lung and digestive carcinoid tumour). Even though other carcinomas, not represented in our series, could benefit from the same approach, such as prostate cancer with the pleural assay of prostate-specific antigen (Cascinu et al, 1997; Brown et al, 1998) or liver cancer with α-fetoprotein (Cascinu et al, 1997), the systematic use of the latter markers is not recommended because these carcinomas are only exceptionally involved in metastatic pleural effusions. On the contrary, lymphomas are more frequently involved but our panel, based on epithelial markers, was inadequate for their diagnosis. Most of the markers were very low in lymphomas (CEA, CA 19–9, CYFRA and SCC) or rarely and moderately increased (CA 15–3). However, two high values of CA 72–4 and one of NSE were observed in patients with lymphomas. Few data concerning lymphomas are available but high values of CA 72–4 have already been described (Ferroni et al, 1990).

Finally, our sensitivities are clearly higher than those previously determined for epithelial malignancies: 73.5% with CEA + CA 72–4 (Ferroni et al, 1990), 71% with CEA and CA 15.3 (Romero et al, 1996) and 78% with CEA + CA 15–3 + CA 72–4 (Villena et al, 1996). Moreover, the sensitivity remained high in cytologically negative effusions since 83.9% of non-lymphomatous effusions were positive with at least one marker of the panel. In the groups of adenocarcinomas and mesotheliomas, very similar sensitivities were observed when all the effusions were considered or only the effusions with a negative cytology. In the groups of small-cell and squamous cell lung carcinomas, not enough patients had effusions with a negative cytology to permit the evaluation of pleural markers in this particular context. However, the sensitivity of the panel was high in cytologically positive effusions and it is highly probable that tumour markers remain efficient in cytologically negative effusions as it was evidenced in adenocarcinomas and mesotheliomas. Assuming that malignancy occurs in about 15% to 25% of pleural effusions (Serre et al, 1990; Fenton and Richardson, 1995; Ferrer et al, 1996; Villena et al, 1996), the negative and positive predictive values for the diagnosis of non-lymphomatous malignant effusions, determined with a theoretical prevalence of 20% and our diagnostic indexes, reach 98.7% and 82.6% respectively. Consequently, this diagnostic approach appears to be of great interest in the event of a negative or a suspicious cytology. It gives an accurate and non-invasive biological criterion to rapidly orient the management of patients towards more invasive procedures such as diagnostic and/or therapeutic thoracoscopy. Markers can also be very useful in patients with a pleurisy that remains idiopathic despite exhaustive evaluations (more than 10% of pleurisies), since these patients are at risk of developing a malignant pleurisy (Leslie and Kinasewitz, 1988; Harris et al, 1995; Ferrer et al, 1996). The cost of these tests is moderate, equivalent to a chest radiography, moreover it can be decreased by using a gradual approach, first assaying the most sensitive markers of the panel.

From a more fundamental point of view, pleural fluid appears as a particularly suitable medium to study the release of tumour markers by cancerous cells. In several patients, very low values of markers were found in serum and extremely high concentrations in pleural fluid (data not shown). One can hypothesize that some cellular clones of the primary tumour able to secrete these tumour markers also developed a high metastatic potential, the tumour marker being involved in the process or not. Moreover, recent studies reported that natural antibodies (Hilgers, 1998) and active specific immunotherapy against tumour markers belonging to the group of epithelial mucins were protective against metastatic progression, patients with a high serum level of mucin before immunotherapy showing a poor prognosis (Maclean et al, 1997). These observations suggest that, beyond its diagnostic interest, pleural assay of some tumour markers could have some prognostic value.

We had hoped that the markers would predict the histological type or even the origin of the tumour. The most convincing results were obtained for the identification of small-cell lung carcinomas in which discriminant analysis showed NSE, SCC and CYFRA to be the most significant markers. The utility of pleural NSE to differentiate non-small-cell lung carcinomas from small-cell lung carcinomas was previously reported (Shimokata et al, 1989). Paone et al (1996) reached the same conclusion by discriminant analysis using

*© 1999 Cancer Research Campaign*

NSE, CEA and tissue polypeptide antigen, a tumour marker corresponding to the assay of cytokeratins 8, 18 and 19 and showing a performance similar to that of CYFRA (Plebani et al, 1995).

Approximately 20% of malignant effusions correspond to metastatic carcinomas of unknown primary site, essentially adenocarcinomas, and pleura account for 10% of the sites of tumour involvement on initial presentation (Lembersky and Thomas, 1996). Therefore, it was of interest to evaluate the prediction of the organ of origin by pleural tumour markers. The 64.8% of correct prediction we obtained did not appear as sufficiently discriminative to orient the search for the primary site. In contradiction with the report of Cascinu et al (1997), which described a high specificity for CA 19–9 and CEA in digestive cancers, our results did not confirm the organ-specificity of the markers. Only the development of more tissue-specific markers will permit the organ of origin of metastatic adenocarcinomas to be efficiently predicted.

In conclusion, from a large series of pleural effusions we demonstrated the high sensitivity and the broad spectrum of the association CEA, CA 15–3, CYFRA and NSE for the diagnosis of malignancy. In pleurisies with a negative cytology, these assays are particularly useful when the clinical presentation cannot clearly exclude an underlying malignancy. Persistent pleurisies also constitute a relevant indication for tumour marker assays. A multicentric and prospective study with this panel would be very helpful to definitively establish the place of these markers in the strategy for the management of pleural effusions. Their putative prognostic value also deserves further investigation.

### ACKNOWLEDGEMENTS

This work was supported in part by grants from the ‘Association pour la Recherche contre le Cancer’. The authors are very grateful to CisBio International France, Abbott France, Boehringer Mannheim France for providing diagnostic kits. The authors also thank J Granié, P Gauthier and H Bagat for their excellent technical assistance.

### REFERENCES

Asseo PP and Tracopoulos GD (1982) Simultaneous enzyme immunoassay of carcinoembryonic antigen in pleural effusion and serum. *Am J Clin Pathol* **77**: 66–71

Brown GA, Ginsberg PC and Harkaway RC (1998) Prostatic adenocarcinoma diagnosed by prostate-specific antigen analysis of pleural fluid. *Urol Int* **60**: 197–198

Callet N, Cohen-Solal Le Nir CC, Berthelot E and Pichon MF (1998) Cancer of the uterine cervix: sensitivity and specificity of serum Cyfra 21.1 determinations. *Eur J Gynaecol Oncol* **19**: 50–56

Cascinu S, Del Ferro E, Barbanti I, Ligi M, Fedeli A and Catalano G (1997) Tumor markers in the diagnosis of malignant serous effusions. *Am J Clin Onco*l **20**: 247–250

Daste G, Serre G, Mauduyt M-A, Vincent C, Caverivière P and Soleilhavoup JP (1991) Immunophenotyping of mesothelial cells and carcinoma cells with monoclonal antibodies to cytokeratins, vimentin, CEA and EMA improves the cytodiagnosis of serous effusions. *Cytopathology* **2**: 19–28

Doweck I, Barak M, Greenberg E, Uri N, Kellner J, Lurie M and Gruener N (1995) Cyfra 21–1. A new potential tumor marker for squamous cell carcinoma of head and neck. *Arch Otolaryngol Head Neck Surg* **121**: 177–181

Fenton KN and Richardson JD (1995) Diagnosis and management of malignant pleural effusions. *Am J Surg* **170**: 69–74

Ferrer JS, Munoz XG, Orriols RM, Light RW and Morell FB (1996) Evolution of idiopathic pleural effusion: a prospective, long-term follow-up study. *Chest* **109**: 1508–1513

Ferroni P, Szpak C, Greiner JW, Simpson JF, Guadagni F, Johnston WW and Colcher D (1990) CA 72–4 radioimmunoassay in the diagnosis of malignant effusions. Comparison of various tumor markers. *Int J Cancer* **46**: 445–451

Garcia-Pachon E, Padilla-Navas I, Dosda MD and Miralles-Llopis A (1997) Elevated level of carcinoembryonic antigen in nonmalignant pleural effusions. *Chest* **111**: 643–647

Grem J (1997) The prognostic importance of tumor markers in adenocarcinomas of the gastrointestinal tract. *Curr Opin Oncol* **9**: 380–387

Guadagni F, Roselli M, Cosimelli M, Ferroni P, Spila A, Cavaliere F, Casaldi V, Wappner G, Abbolito MR and Greiner JW (1995) CA 72–4 serum marker – a new tool in the management of carcinoma patients. *Cancer Invest* **13**: 227–238

Hanley JA and McNeil BJ (1983) A method of comparing the areas under receiver operating characteristic curves derived from the same cases. *Radiology* **148**: 839–843

Harris RJ, Kavuru MS, Rice TW and Kirby TJ (1995) The diagnostic and therapeutic utility of thoracoscopy. *Chest* **108**: 828–841

Hilgers JH (1998) Natural antibodies against MUC1 protect against disease progression in early breast cancer. *Tumor Biol* **19** (S2)

Klockars M, Lindgren J, Pettersson T, Hellstrom PE and Norhagen A (1980) Carcinoembryonic antigen in pleural effusions: a diagnostic and prognostic indicator. *Eur J Cancer* **16**: 1149–1152

Lembersky BC and Thomas LC (1996) Metastases of unknown primary site. *Med Clin North Am* **80**: 153–171

Leslie WK and Kinasewitz GT (1988) Clinical characteristics of the patient with nonspecific pleuritis. *Chest* **94**: 603–608

Loddenkemper R and Boutin C (1993) Thoracoscopy: present diagnostic and therapeutic indications. *Eur Resp* J **6**: 1544–1555

MacLean GD, Reddish MA and Longenecker BM (1997) Prognostic significance of preimmunotherapy serum CA 27.29 (MUC-1) mucin level after active specific immunotherapy of metastatic adenocarcinoma patients. *J Immunother* **20**: 70–78

Menard O, Dousset B, Jacob C and Martinet Y (1993) Improvement of the diagnosis of the cause of pleural effusion in patients with lung cancer by simultaneous quantification of carcinoembryonic antigen (CEA) and neuron-specific enolase (NSE) pleural levels. *Eur J Cancer* **29A**: 1806–1809

Mezger J, Calavrezos A, Drings P, Gatzemeier U, Kaukel E, Konietzko N, Koschel G, Lamerz R, Von Pawel J and Romer W (1994) Value of serum and effusion fluid CEA levels for distinguishing between diffuse malignant mesothelioma and carcinomatous pleural metastases. *Lung* **172**: 183–184

Morita T, Kikuchi T, Hashimoto S, Kobayashi Y and Tokue A (1997) Cytokeratin-19 fragment (CYFRA 21–1) in bladder cancer. *Eur Urol* **32**: 237–244

Niklinski J and Furman M (1995) Clinical tumour markers in lung cancer. *Eur J Cancer Prev* **4**: 129–138

Nyberg P, Soderblom T, Pettersson T, Riska H, Klockars M and Linko L (1996) Neurone-specific enolase levels in pleural effusions in patients with rheumatoid arthritis. *Thorax* **51**: 92–94

Paone G, De Angelis G, Greco S, Fiorucci F, Bisetti A and Ameglio F (1996) Carcinoembryonic antigen, tissue polypeptide antigen and neuron-specific enolase pleural levels used to classify small-cell and non-small-cell lung cancer patients by discriminant analysis. *J Cancer Res Clin Oncol* **122**: 499–503

Peto J, Hodgson JT, Matthews FE and Jones JR (1995) Continuing increase in mesothelioma mortality in Britain. *Lancet* **345**: 535–539

Plebani M, Basso D, Navaglia F, De Paoli M, Tommasini A and Cipriani A (1995) Clinical evaluation of seven tumour markers in lung cancer diagnosis: can any combination improve the results? *Br J Cancer* **72**: 170–173

Pujol JL, Grenier J, Daures JP, Daver A, Pujol H and Michel FB (1993) Serum fragment of cytokeratin subunit 19 measured by CYFRA 21–1 immunoradiometric assay as a marker of lung cancer. *Cancer Res* **53**: 61–66

Rapellino M, Pecchio F, Piantino P and Cellerino A (1990) Tumor markers determination in extrahematic fluids. *J Nucl Med Allied Sci* **34**: 128–136

Rittgers RA, Loewenstein MS, Feinerman AE, Kupchik HZ, Marcel BR, Koff RS and Zamcheck N (1978) Carcinoembryonic antigen levels in benign and malignant pleural effusions. *Ann Intern Med* **88**: 631–634

Romero S, Fernandez C, Arriero JM, Espasa A, Candela A, Martin C and Sanchez-Paya J (1996) CEA, CA 15–3 and CYFRA 21–1 in serum and pleural fluid of patients with pleural effusions. *Eur Respir* J **9**: 17–23

Sahn SA (1988) The pleura. *Am Rev Respir Dis* **138**: 184–234

Salama G, Miédougé M, Rouzaud P, Mauduyt M-A, Pujazon M-C, Vincent C, Carles P and Serre G (1998) Evaluation of pleural CYFRA 21–1 and carcinoembryonic antigen in the diagnosis of malignant pleural effusions. *Br J Cancer* **77**: 472–476

Satoh H, Sumi M, Yagyu H, Ishikawa H, Suyama T, Naitoh T, Saitoh T and Hasegawa S (1995) Clinical evaluation of CYFRA 21–1 in malignant pleural fluids. *Oncology* **52**: 211–214

*© 1999 Cancer Research Campaign*

Serre G, Daste G, Vincent C, Mauduyt M-A and Soleilhavoup JP (1990) Diagnostic approach to the patient with pleural effusion: cytologic analysis of pleural fluid. In: *International Trends in General Thoracic Surgery*, Vol. 6, Deslauriers J and Lacquet LK (eds), pp. 35–45. CV Mosby: St Louis

Shimokata K, Niwa Y, Yamamoto M, Sasou H and Morishita M (1989) Pleural fluid neuron-specific enolase. A useful diagnostic marker for small cell lung cancer pleuritis. *Chest* **95**: 602–603

Soletormos G, Nielsen D, Schioler V, Skovsgaard T and Dombernowsky P (1996) Tumor markers cancer antigen 15.3, carcinoembryonic antigen, and tissue polypeptide antigen for monitoring metastatic breast cancer during first-line chemotherapy and follow-up. *Clin Chem* **42**: 564–575

Toumbis M, Rasidakis A, Passalidou E, Kalomenidis J, Alchanatis M, Orphanidou D and Jordanoglou J (1996) Evaluation of CYFRA 21–1 in malignant and benign pleural effusions. *Anticancer Res* **16**: 2101–2104

Villena V, Lopez-Encuentra A, Echave-Sustaeta J, Martin-Escribano P, Ortuno-de-Solo B and Estenoz-Alfaro J (1996) Diagnostic value of CA 72–4, carcinoembryonic antigen, CA 15–3, and CA 19–9 assay in pleural fluid. A study of 207 patients. *Cancer* **78**: 736–740

Villena V, Lopez Encuentra A and Rodriguez FP (1998) False-positive results of carcinoembryonic antigen in pleural effusions. *Chest* **113**: 1143–1144

Zeimet AG, Marth C, Offner FA, Obrist P, Uhl-Steidl M, Feichtinger H, Stadlmann S, Daxenbichler G and Dapunt O (1996) Human peritoneal mesothelial cells are more potent than ovarian cancer cells in producing tumor marker CA-125. *Gynecol Oncol* **62**: 384–389

*© 1999 Cancer Research Campaign*
